# Effects of cannabis regulation in Switzerland: Study protocol of a randomized controlled trial

**DOI:** 10.3389/fpsyt.2023.1139325

**Published:** 2023-03-23

**Authors:** Lavinia Baltes-Flueckiger, Regine Steinauer, Maximilian Meyer, Marc Vogel, Marc Walter

**Affiliations:** ^1^Psychiatric and Psychotherapeutic Clinic, Psychiatric Services Aargau, Windisch, Switzerland; ^2^Health Department of Canton Basel-Stadt, Basel, Switzerland; ^3^Psychiatric University Clinics Basel, University of Basel, Basel, Switzerland; ^4^Faculty of Medicine, University of Basel, Basel, Switzerland

**Keywords:** recreational cannabis regulation, cannabis use disorder, mental disorders, physical health, study protocol, randomized controlled trial

## Abstract

**Background:**

Cannabis is the most widely used illicit substance. Various countries have legalized cannabis for recreational use. Evidence on the health effects of cannabis regulation remains unclear and is mainly based on observational studies. To date, there is no randomized controlled study evaluating the impact of cannabis regulation for recreational use compared to the illicit market on relevant health indicators. The present study (“Weed Care”) is the first to evaluate the impact of regulated cannabis access in pharmacies versus a waiting list control group representing the illicit market on problematic cannabis use as well as on mental and physical health.

**Methods:**

The study is divided into two parts—a randomized controlled study of 6 months followed by an observational study of 2 years. Participants (*N* = 374) are randomly assigned to either the experimental group with access to legal cannabis in pharmacies or to the waiting list control group representing the current legal framework in Switzerland, namely the illicit market. After 6 months, all participants will have access to legal cannabis for the following 2 years (observational study). The primary outcome is problematic cannabis use as measured with the Cannabis Use Disorders Identification Test-Revised (CUDIT-R). Secondary outcomes are cannabis use patterns, mental disorders (e.g., depression, anxiety, and psychosis) and physical health (e.g., respiratory symptoms). Primary and secondary outcomes will be assessed online every 6 months. The study is approved by the responsible ethics committee as well as by the Swiss Federal Office of Public Health.

**Discussion:**

Findings from this study may provide a scientific basis for future discussions about addiction medicine and cannabis policy in Switzerland.

**Clinical Trial Registration:**

ClinicalTrials.gov (NCT05522205). https://clinicaltrials.gov/ct2/show/NCT05522205

## Introduction

Cannabis has been an illicit substance in countries that signed the UN Single Convention for more than 50 years. Nevertheless, cannabis is the most commonly used illicit substance globally. In 2020, the United Nations estimated that 209 million or 4% of persons of the global adult population had used cannabis in the previous year (1). The last-year prevalence of the European adult population is 22.2 million or 7.7% (2). Switzerland has one of the highest national prevalence rates of cannabis use in Europe. Whereas the lifetime prevalence for European adult population is 27.3%, the lifetime prevalence for the Swiss adult population is 34% (3). Approximately 4% of the Swiss population used cannabis in the past-month (3).

These prevalence rates suggest that cannabis prohibition has not been able to sustainably reduce cannabis use. In contrast, prohibition is associated with high health and social costs due to unsafe product quality, stigmatization and criminalization of users as well as access barriers to harm reduction, prevention, and treatment (4, 5). Furthermore, cannabis causes less societal and individual harm compared to illicit opioids and stimulants (6) or legal drugs such as alcohol and tobacco (7, 8). The comparatively high number of users, the modest harms of cannabis use, as well as the high costs of its prohibition have led to increased public discourse reconsidering prohibition and prompting alternative cannabis regulation models.

Besides strict prohibition, the opposite pole of the legislative spectrum—free commercial market—is equally associated with increased public health costs, because industry is interested in promoting cannabis use (9). In contrast, the middle-ground regulation may minimize the adverse effects of both extremes. This could be achieved by implementing policies to improve public health and safety, to protect minors, as well as to reduce crime (9, 10). These policies may include, e.g., pricing according to product potency, a governmental monopoly on production and sales, restriction of advertisement, minimum age, health education on safer use, prevention programs, and treatment-referrals (11).

Various states have introduced legal cannabis laws for recreational use and further countries propose to legalize cannabis in the near future. The implemented regulatory models vary widely. In particular, since 2012 over a dozen US states have implemented a commercial cannabis model similar to the alcohol and tobacco regulations (12). Canada followed suit in 2018 with a focus on public health (13), while in Uruguay cannabis is strictly regulated and controlled by the government since 2013 (14). Given the general shift in cannabis legislation—away from prohibition toward regulation—knowing the effects of these policies on cannabis use patterns as well as on mental and physical health is paramount for clinicians and policymakers alike. However, evidence for public health from available observational studies is inconclusive.

Recreational cannabis legalization in US states has been associated with an increased frequency of cannabis use among adults (15, 16). Among adolescents and young adults (< 21 years), there was no association between cannabis legalization and cannabis use (15, 17–19). In Uruguay, the legalization of recreational cannabis was not associated with past-month- and past-year-prevalence among young adults (18–21 years); however, it was associated with a transitory increase in risky and more frequent use in young adults that decreased thereafter (20). The cannabis regulation in Canada was associated with an increased 3-month prevalence among adults (> 25 years), but not related with increased frequency of use (21, 22). Additionally, a shift in mode of administration toward lower-risk methods has been observed. Specifically, cannabis users reported less cannabis smoking, increased vaporizing, and consumption of edibles following cannabis legalization (21).

To assess the public health impacts of recreational cannabis regulation, previous studies predominantly evaluated the prevalence and frequency of cannabis use (23). However, these outcomes are of limited clinical significance since the majority of cannabis users are low-risk users and do not suffer from adverse health outcomes related to their use (24). Nonetheless, between 10 and 30% of those, who use cannabis, do fulfill the criteria for a cannabis use disorder (25–28). As cannabis use disorders substantially contribute to the global disease burden (29), investigating the impact of legalization on cannabis use disorder may be of greater concern for public health.

Additionally, not only cannabis use, but also comorbid mental and physical health indicators are of major interest for public health and drug policy reforms (23). Cannabis use disorder has been associated with comorbid psychiatric disorders (30, 31), in particular with an increased risk of use disorders of other substances, psychosis (32, 33), anxiety (34), and depression (35, 36). Whereas extensive evidence has shown that frequent use of potent cannabis is related to an increased risk of psychosis, the evidence for depression and anxiety is less clear (37–39). However, a causal relation was not established in these studies. Thus, it remains unclear whether the increased risk of mental disorders is attributable to cannabis use or other confounding factors. Furthermore, the relation between cannabis use and physical health is also uncertain. Chronic bronchitis is the most consistently found adverse effect of cannabis smoking (40, 41). In contrast to the adverse health effects of cannabis use, there is additional growing evidence evaluating the beneficial health effects of medical cannabis. Medical cannabis has been associated with improvements of symptoms of mental disorders, including anxiety, schizophrenia, and sleep (42, 43). Further, low-to-moderate evidence supports the treatment of a range of physical conditions, including chronic pain, nausea, and vomiting caused by chemotherapy, and some treatment resistant epilepsies (44, 45). However, evidence on the impact of recreational cannabis legalization on comorbid mental and physical health outcomes in cannabis users is rare. One epidemiological study has shown that cannabis laws for medical use in the US are associated with reduced mental health problems (46). Research on the impacts of recreational cannabis laws on mental and physical health indicators in cannabis users is yet lacking.

To our knowledge, there is no randomized controlled study evaluating the effects of recreational cannabis legalization compared to the illicit market on relevant health outcomes. Available evidence is based on observational studies, including pre-post-legalization comparisons or comparisons between states with recreational cannabis regulation and states without. However, these study designs are not able to distinguish between secular trends and changes due to cannabis regulation and thus, causality remains unclear (47). The aim of the present randomized controlled study is to investigate the effects of cannabis regulation for recreational purposes compared to the illicit market on problematic cannabis use, mental disorders, and physical health. Although cannabis for recreational use is prohibited in Switzerland, scientific studies on recreational cannabis are allowed since 2021 (48). Hence, Switzerland offers a unique opportunity to gain scientific knowledge about alternative cannabis regulation approaches. We hypothesize that regulated cannabis access in pharmacies will reduce problematic cannabis use as within a regulated approach several conditions are available to facilitate lower-risk use and treatment. Furthermore, we hypothesize that regulated cannabis access will improve mental and physical health outcomes.

Moreover, the effects of chronic cannabis use on epigenetic DNA-expression regulation are not yet fully understood. Its mechanisms may provide explanations for the development of cannabis use disorder and cannabis withdrawal syndrome, as well as emotion-regulating effects of cannabis use. To date, an association between cannabis use and the expression of neurotrophic growth factors (49, 50) as well as changes in the hypothalamic–pituitary–adrenal (HPA) axis (51) have been observed. The Pro-opiomelanocortin (POMC) and NR3C1 genes play an important role in the regulation of the HPA axis, through which the release of stress hormones like adrenocorticotropin (ACTH) and cortisol occurs. The NR3C1 gene encodes the glucocorticoid receptor to which glucocorticoids such as dexamethasone bind and is essential for further signal transduction (52). POMC is a hormone precursor of interest, as it is susceptible to epigenetic regulation through DNA methylation as well (53). In patients with alcohol and opioid dependence, epigenetic gene express regulation mechanisms are associated with craving symptoms (54, 55). A pilot study showed a significant increase in POMC methylation in opioid-dependent patients following heroin injection (52). The neurotrophic growth factor brain-derived neurotrophic factor (BDNF) is associated with central dopamine release. Hence, an alteration in BDNF expression may be related to the reward effects and dependence potential of psychotropic substances (56). In the context of neurocognitive performance, as well as the development of substance-related dependence disorders, nerve growth factor (NGF), vascular endothelial growth factor-A (VEGF-A), and glial cell line-derived neurotrophic factor (GDNF) have been investigated in addition to BDNF (52, 57–59). However, in connection with cannabis use, the epigenetic regulation of these genes has so far been insufficiently studied and research on non-clinical populations of cannabis users is scarce.

## Methods

### Design

The study “Weed Care” is designed as a randomized, controlled, unblinded superiority trial with two parallel groups. Participants will be randomized to the experimental group with legal cannabis access in pharmacies or to the waiting list control group representing the illicit market. The primary endpoint is 6 months of regulated cannabis access. After 6 months, an observational study of 2 years will follow. During this time, both groups will have access to cannabis in pharmacies. This monocenter study will be conducted in the canton Basel-Stadt in Switzerland. The study has been approved by the local ethics committee “Ethikkommission Nordwest- und Zentralschweiz” (EKNZ) as well as by the Swiss Federal Office of Public Health. Furthermore, the study is designed in agreement with the Declaration of Helsinki, the ICH-GCP guidelines, and the Swiss law, notably the amendment of the Federal Act on Narcotics and Psychotropic Substances. The latter defines certain conditions for scientific studies with recreational cannabis such as minimum age of participants and maximum tetrahydrocannabinol (THC-) content (48).

### Eligibility criteria

Participants must provide written, informed consent before any study procedures occur. Eligibility criteria are checked by the study physician. Participants eligible for the study must comply with all of the following inclusion criteria:

- Minimum age of 18 years
- Cannabis use at least once per month during the last 6 months (including a positive THC-urine sample)
- Sufficient German language skills
- Residence in Basel-Stadt
- Internet access in order to answer online-questionnaires.

Exclusion criteria include:

- Current pregnancy or breast feeding
- Current inpatient psychiatric treatment
- Acute psychosis
- Acute suicidality
- Severe cognitive impairment that does not allow to understand the study information nor to follow the study procedure
- Any intention to move away from Basel-Stadt within the next 12 months.

### Recruitment

A media conference in September 2022 informed the public about the current study. From this point on, interested persons were able to register online. After online registration, potential participants are contacted by phone for a first screening by the study nurse. Following, they are invited for a face-to-face interview with the study physician. In this interview, detailed information about the study is provided and written informed consent is obtained following the evaluation of inclusion/exclusion criteria. Individuals suitable for participation are randomly assigned to the experimental or control group. Furthermore, each participant will be asked to provide a blood sample. Recruitment has begun in September 2022 and will last until the required number of participants (*N* = 374) is reached, but no longer than 1 year.

### Participant timeline

**Figure 1:**
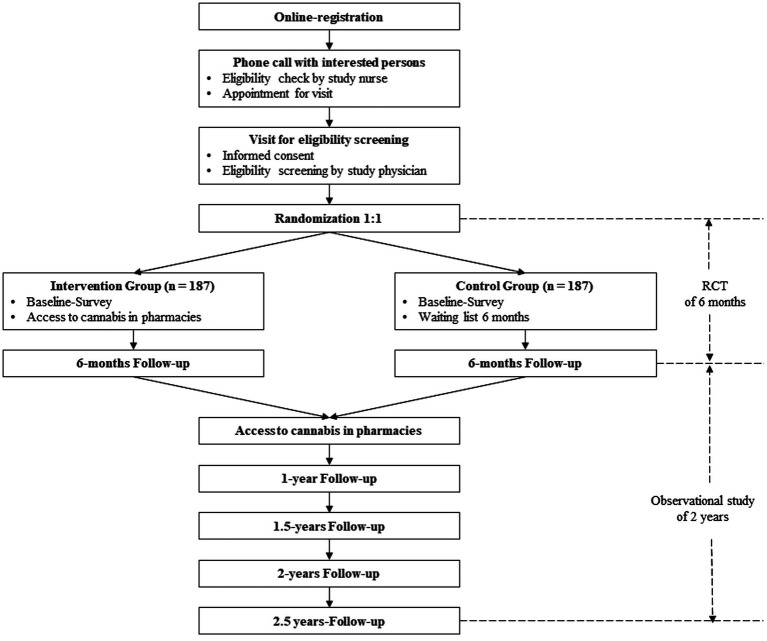
Flowchart of the study.

Figure 1 shows the participant flow through the study. Legal cannabis access in pharmacies started on January 30, 2023. Both groups—the experimental as well as the control group—were invited to answer the baseline-survey two weeks prior to cannabis access (see Table 1). Follow-up assessments, using the full battery, will be collected every 6 months from baseline (at 6, 12, 18, 24, and 30 months) with a total of five main follow-up assessments. Additionally, short follow-up assessments are conducted every 2 months (at 2, 4, 8, 10, 14, 16, 20, 22, 26, and 28 months) with a total of ten short follow-up assessments. Main assessments lasts approximately 30 min, short assessments 10 min. Participants are required to answer the main assessments within 2 weeks, in order to buy cannabis in pharmacies. If they do not answer the questionnaires, they will be blocked.

**Table 1:** Overview of all measures and their assessment times.

Measure	Target	Baseline	Every 2 months follow-up-	Every 6 months follow-up	2.5 years follow-up
Demographics	Sociodemographic data	X			X
CUDIT-R	Problematic cannabis use	X		X	X
CS-CCU*	Cannabis use patterns	X	X	X	X
LRCU*	Lower-risk cannabis use	X		X	X
Motives*	Cannabis use motives	X		X	X
PANAS	Positive and negative affect	X	X	X	X
PHQ-9	Depression	X		X	X
GAD-7	Anxiety	X		X	X
ASRS-V1.1	Attention deficit and hyperactivity	X		X	X
ERIRaos	Psychosis	X		X	X
SCL-90 Somatization	Physical symptoms	X		X	X
CAT	Respiratory symptoms	X		X	X
EuroQol-5D	Quality of life	X		X	X
SAE*	Serious adverse events	X	X	X	X
PSQI	Sleep	X		X	X
GLTEQ	Physical activity	X		X	X
AUDIT-C	Alcohol use	X		X	X
ASSIST	Illicit substance use	X		X	X
S-CNU*	Nicotine use	X		X	X
Drugs	Drug use	X		X	X
Blood sample	Epigenetic analysis	X			

* These are no official instruments. These questionnaires were developed in cooperation with scientific partners from the universities of Basel, Bern, Geneva, Lausanne, St. Gallen and Zürich as well as the Swiss Federal Office of Public Health as a common basis for data collection of the pilot trials in Switzerland.

ASRS-V1.1 Adult ADHD Self-Report Scale, ASSIST Alcohol, Smoking and Substance Involvement Screening Test, AUDIT-C Alcohol Use Disorders Identification Test, CAT COPD Assessment Test, CS-CCU Comprehensive Short Questionnaire of Current Cannabis Use, CUDIT-R Cannabis Use Disorders Identification Test—Revised, EuroQol-5D European Quality of Life, ERIraos Early Recognition Inventory Checklist, GAD-7 Generalized Anxiety Disorder, GLTEQ Godin Leisure Time Exercise Questionnaire, LRCU Lower-Risk Cannabis Use, PANAS Positive Affect and Negative Affect Scale, PHQ-9 Patient Health Questionnaire, PSQI Pittsburgh Sleep Quality Index, SAE Serious Adverse Events, SCL-90 Somatization Symptom Checklist-90, S-CNU Short Questionnaire of Current Nicotine Use.

### Interventions

Eligible participants are randomized in equal proportions between the experimental and the control group. The experimental group has access to regulated cannabis in nine pharmacies that have been previously selected. The control group will have access to regulated cannabis with 6-month delay. After these 6 months, both groups will have access to cannabis in pharmacies for the following two years. Several potential conditions are available to facilitate lower-risk cannabis use and treatment within the intervention of cannabis regulation (60, 61).

- Product quality: Participants are provided high quality cannabis products without any contamination and with declared THC/Cannabidiol (CBD) content. Participants can choose between six different cannabis products that are produced by a Swiss cannabis company: Four flower products and two hash products with different THC and CBD ratios.
- Regulated access: THC content is limited to a maximum of 20% THC. Additionally, the cannabis amount that participants can buy per month is limited to up to 10 g of total THC per month (participants can buy more of a product with low THC content compared to a product with high THC content).
- Pricing: Prices of the cannabis products in pharmacies are comparable to the products on the illicit market, so they do not compete. In order to promote lower-risk products, products with higher THC content are more expensive than products with lower THC content.
- Protection of minors: Only persons older than 18 years are allowed to participate in the study.

Facilitated access to users:

- Prevention: Pharmacists inform participants about lower-risk use guidelines such as products with lower THC content or vaporizers (62, 63). Furthermore, health prevention information and lower-risk use guidelines will be also available on the product packaging as well as on the study website.
- Early detection, early intervention, and encouraging treatment: Pharmacists may notice any potential health risks in participants. In this case, pharmacists will conduct a brief intervention and will recommend to contact the study physician, a local counseling or treatment institution. In case of a severe health risk, pharmacists may impose a consultation with the study physician. He will check whether legal cannabis access is still medically acceptable for this participant. The study physician trained all pharmacists on lower-risk cannabis guidelines as well as on red flags of cannabis use disorder, psychosis, depression, and suicidality. Besides the pharmacists, cannabis use disorder, depression, anxiety, and psychosis will be assessed every 6 months *via* online-assessments. If participants show a significant deterioration in one of these aspects, the study physician will contact the affected participant, will evaluate whether cannabis access remains medically responsible, and will provide treatment options.

The control group shall display the status quo in Switzerland, namely the situation of the illicit market. Therefore, during the first 6 months, the waiting list control group has no access to cannabis in pharmacies. They have to continue to obtain their cannabis *via* their usual sources. Additionally, any prevention information and early detection and intervention in pharmacies are lapsed for this group during the first 6 months.

### Outcomes

Table 1 provides an overview of all measures and time points when they are assessed in the study. All questionnaires are administered in German and all available German versions of questionnaires are used. Additionally, all measurements are self-reported and assessed online.

#### Primary outcome

The present study evaluates whether regulated cannabis access improves problematic cannabis use. The primary outcome is the difference of problematic cannabis use between the two groups after 6 months. Problematic cannabis use will be assessed by the “Cannabis Use Disorders Identification Test-Revised (CUDIT-R)” (64). The scale includes eight statements about cannabis use with answers ranging from “never” (0) to “daily or almost daily” (4). The total score ranges from 0 to 32 with scores of 13 and more indicating a possible cannabis use disorder. Problematic cannabis use assessed with CUDIT-R could be reduced after 6 months (65). In order to detect a change in cannabis use as well as to keep the waiting period for the control group as short as possible, the trial period is set at 6 months.

#### Secondary outcomes

##### Cannabis use patterns

- The *Comprehensive Short Questionnaire of Current Cannabis Use* (CS-CCU) evaluates cannabis use patterns during the last 30 days. The questionnaire includes 23 questions about cannabis quantity used, frequency, products, and mode of administration. Moreover, participants will be asked whether they continue to obtain illegal cannabis. Specifically, the amount of illegal cannabis and the frequency of its use during the last 30 days are assessed. In addition, participants’ satisfaction of the regulated cannabis access will be assessed (e.g., products, pharmacies, and information provided).
- *Cannabis consumption motives* are assessed with ten items covering a range of motives such as “to relax,” “to reduce physical symptoms,” and “to enhance concentration.” Ten statements will be rated on a 5 point-Likert scale from “never/almost never” (0) to “almost always/always” (4).
- To assess knowledge and behavior of safer cannabis use guidelines, the scale *Lower-Risk Cannabis Use–Knowledge and Behavior* is used. This measure includes 24 statements that must be answered on a 4 point-Likert scale that ranges from 0 to 4. The total score ranges from 0 to 48 with higher scores indicating more knowledge and behavior of lower-risk cannabis use.

Additional objective data on cannabis purchased are collected in pharmacies. These data include type of products (THC- and CBD-content) purchased and quantity purchased per month.

##### Mental health

- The *Patient Health Questionnaire for Depression (PHQ-9)* includes nine items that assess the degree of depression. Answers range from “not at all” (0) to “almost daily” (3) with a total score between 0 and 27. A score of 10 or higher is considered indicative of a major depressive disorder (66, 67).
- The *Generalized Anxiety Disorder Scale-7 (GAD-7)* is an instrument that consists of seven items on anxiety symptoms with answers ranging from “not at all” (0) to “almost daily” (3) (68). The total scores are between 0 and 21 and scores of 10 or above are considered indicative of a generalized anxiety disorder (69). GAD-7 showed to be a valid and reliable measure for anxiety (69).
- The *Adult-ADHD Self-Report Scale (ASRS-V1.1)* is a six-item questionnaire on attention deficit and hyperactivity disorder (ADHD) with answers ranging from “never” to “very often.” Total scores of four or above are considered indicating a positive screening result of ADHD (70).
- The Early Recognition Inventory (ERIraos) Checklist is a screening instrument for the early recognition of psychosis risk (71). We only use the items on early psychosis including eight statements that are answered with “no” (0) / “yes” (1). Scores of one ore higher are considered as indicative for an early psychosis.
- The *Positive and Negative Affect Schedule (PANAS)* is a scale to assess positive and negative effects in the last days, that addresses affective states (72, 73). The measure includes 20 items that are rated on a 5 point-Likert scale ranging from “very slightly or not at all” (1) to “extremely” (5). Mean score of each dimension (positive and negative affect) indicates a higher positive and negative affective state, respectively.

##### Physical health

- The *EuroQuol-5D* contains five dimensions of health-related quality of life (morbidity, self-care, daily activities, pain/discomfort, and depression/anxiety) rated on a scale ranging from “no problems” (0) to “extreme problems” (5). Furthermore, participants’ health status is assessed by a single index measure ranging from 0 to 100 (74). The total score of the five dimensions is used as a measure of quality of life.
- Physical symptoms are assessed with the dimension *Somatization* of the *Symptom Checklist-90 (SCL-90)* (75). This subscale consists of 12 items that are scored on a 5 point-Likert scale ranging from “not at all” (1) to “extremely” (5). The total score ranges from 12 to 60 and higher scores indicate higher physical discomfort.
- Respiratory symptoms are evaluated with the *COPD Assessment Test (CAT)*. This is an eight-item scale covering all aspects of chronic obstructive pulmonary disease (COPD) and indicates the severity of COPD. Each item is formatted as a semantic six-point differential scale. Higher total score indicates worse respiratory health status (76).
- *Serious adverse events* are assessed with two items asking participants whether they were hospitalized longer than 24 h and whether they experienced a life-threatening event since the last survey.

##### Consumption of other substances

- The *Alcohol Use Disorders Identification Test-Consumption (AUDIT-C)* is a screening tool including three items that assess problematic and risky alcohol use. The questions are answered on a 5 point-Likert scale ranging from 0 to 4 with a total score between 0 and 12. Scores of 2 or above in women and of 4 or above in men are considered as indicative for alcohol misuse (77).
- The *Alcohol, Smoking and Substance Involvement Screening Test (ASSIST)* is an instrument that assesses drug consumption with answers ranging from “never” (0) to “daily or almost daily” (4) (78). We only use the pre-screener question without the items on tobacco and alcohol and with an adapted time frame to 6 months instead of 3 months.
- The *Short Questionnaire of Current Nicotine Use (S-CNU)* includes nine items about current nicotine use. Additionally, the two items of Heaviness of Smoking Index are included (79). In total, the instrument consists of 11 items.
- *Medication use* is assessed with a ten-item scale covering ten medications with answers ranging from “never” (0) to “almost daily/daily” (4).

##### Health behaviors

- Sleep is measured with the two items from the *Pittsburgh Sleep Quality Index* that have been shown the highest correlation between single-item and the final score of the full scale (single-item—total correlation), *r* = 0.83 for sleep quality and *r* = 0.80 for sleep duration (80). Participants rate their sleep quality on a 4point-Likert scale from “very bad” (1) to “very good” (4) and indicate the hours of actual sleep.
- Physical activity is evaluated with the *Godin Leisure Time Exercise Questionnaire.* Participants will be instructed to indicate the number of minutes engaged in mild, moderate, and strenuous exercise during the last week. The minutes of mild, moderate, and strenuous exercise are weighted by metabolic equivalents and then summed to produce a total weekly leisure activity score. Higher scores reflect higher levels of physical activity (81).

Besides the current study, further studies on cannabis regulation in Switzerland are planned in order to gain insights into different regulation models and its impact on health, cannabis use, and safety, among others. The Swiss Federal Office of Public Health is interested in conducting analyses over all these studies in order to provide a more broadly evidence-based information for policymakers. Therefore, the aim is that these studies include a minimum set of the same questionnaires that allows to compare the data of the various studies. This set of questionnaires was developed by researchers of several Swiss universities that are involved in these studies and the Swiss Federal Office of Public Health. This set includes questionnaires about cannabis use patterns, problematic cannabis use, mental and physical health, and consumption of other substances. These questionnaires are also included in the present study.

##### Epigenetic analysis

At baseline, a blood sample will be collected from participants for analysis of epigenetic gene express regulation. DNA methylation of genes regulating the HPA (POMC, and NR3C1) as well as neurotrophic growth factors (BDNF, GDNF, NGF, and VEGF-A) will be investigated.

### Sample size

The sample size was calculated on the basis of the primary hypothesis. A reliable change in problematic cannabis use is defined as a change of two points or greater in mean CUDIT-R after 6 months (65). The power analysis (power of 0.8, alpha of 0.05, dropout of 20%) identified a target enrollment of 374 cannabis users—187 per group—to detect a reliable change in problematic cannabis use after 6 months.

### Statistical methods

Descriptive statistics will be reported for sociodemographic and baseline characteristics. For the analysis of the primary outcome, analysis of covariance will be used (82). This model includes CUDIT-R mean after 6 months as dependent variable and the study group (experimental, control group) and the CUDIT-R mean at baseline as independent variable. Linear mixed models will be conducted for the analyses of the secondary outcomes in order to evaluate between-person and within-person differences during the 2 years of regulated cannabis access. Additionally, potential effect modification will be analyzed (gender, age, education, and cannabis consumption motives). Data will be analyzed according to the intention-to-treat-principle and thus (82), all patients being enrolled in the study and answered the baseline survey will be included.

### Data collection

All data of main- and short-assessments are self-reported data and will be collected and stored in the Research Electronic Data Capture (REDCap) software (83) at the University Hospital Basel. With the exception of the eligibility screening, participants will enter their own data online. Eligibility screening data is entered by the study nurse. Randomization of participants to either control or experimental group with a 1:1 allocation is also conducted in REDCap.

Cannabis sales data in pharmacies will be collected in a separate software named Cannavigia that traces the entire cannabis supply network (84). Participants are registered in Cannavigia and receive a unique identifier to ensure data collection to be as anonymous as possible. A separate list of identifiers linked to personal information is only accessible for study physicians and study nurses. Datasets of both softwares will be used for analyses.

### Data monitoring and auditing

A risk-based monitoring will be conducted to ensure the quality control. Based on the “Guidelines for risk-based monitoring,” the risk was estimated low (85). The study investigators conducted an initiation visit at the study center before eligibility screenings have started. Additionally, they will monitor the survey data in REDCap on a regular basis. Furthermore, a monitor, who is independent of the study organizers, will conduct three visits in four randomly selected pharmacies in order to ensure comparable study procedure in the pharmacies. The first visit will be conducted after cannabis access has started, the second one after 6 months, and the third after the final cannabis product was sold.

### Safety

We do not expect increased health risks for participants, since participants must already be regular cannabis users prior to study participation. In case of serious adverse events (SAE), the research team will be notified. Additionally, participants will be asked in every online-assessment whether they have experienced a SAE since the last assessment (life-threatening event, hospitalization longer than 24 h). The relation of any SAE to study procedures will be investigated and reported to the local ethics committee.

## Discussion

Various states have legalized cannabis for recreational use and further countries propose to legalize cannabis in the near future. Implemented regulatory approaches vary widely ranging from commercial market to strict regulation by the government (11). Existing evidence on the public health impact of recreational cannabis regulation is contradictory and inconclusive. Some studies have shown an association between the regulation of cannabis for recreational use and increased frequency of cannabis use among adults (15, 16), whereas others have shown no relation or a relation with increased three-month prevalence (21, 22). A recent systematic review has suggested that past-month cannabis use in adults (> 26 years) has increased after recreational cannabis legalization, whereas young adults (18–26 years) and adolescents (12–17 years) did not show an increase in past-month cannabis use (86). However, existing evidence is mainly based on observational data evaluating pre-post-legalization or comparisons between states with and without cannabis legalization. These study designs do not allow to derive causal relations. Furthermore, evidence on the impact of cannabis regulation on mental and physical health is largely lacking. Hence, there is still considerable uncertainty regarding the positive and negative health effects of cannabis regulation. Scientific evidence on policy options and their public health impact is needed.

In Switzerland, cannabis for recreational use is prohibited, but the Federal Act on Narcotics and Psychotropic Substances allows scientific studies with cannabis since 2021 (48). The aim of this law is to increase evidence-based knowledge of the advantages and disadvantages of different regulatory approaches, and thus, to provide a scientific basis for discussions about future cannabis policy in Switzerland. To our knowledge, the present study “Weed Care” is the first randomized controlled trial that evaluates the effects of recreational cannabis regulation compared to the illicit market on problematic cannabis use as well as on mental and physical health outcomes. This study design will provide compelling evidence elucidating benefits and harms of recreational cannabis regulation on relevant health indicators. For example, if results show that cannabis use and health outcomes improve after 6 months in the group with legal access compared to the control group, policymakers may consider the evaluated type of cannabis regulation to be superior to the prohibition. Besides improved users’ health, an additional benefit might be decreasing health costs in the long-term. However, this is not part of the present study. If results show no difference in health outcomes between the two groups, the analyzed regulation model is not inferior to prohibition and may still display a potential alternative approach for drug policy since prohibition has not achieved its aims over the last decades. In contrast, if results show that cannabis use and health outcomes deteriorate in the group with legal cannabis access compared to the control group, policymakers may draw the conclusion that these results favors cannabis prohibition. However, all conclusions should be drawn with caution, since the present study investigates one potential regulation model and its effect on health outcomes. Further research is needed to investigate the effects of regulated cannabis on additional outcomes such as on youth, criminality, stigmatization, or illicit market. Moreover, the evaluation of other regulation models such as cannabis social clubs or cannabis shops is substantial in order to compare the benefits and harms of different regulation approaches.

## Limitations

The study shows several limitations. First, the generalizability of results is limited. Certain groups may not participate in the study due to specific circumstances such as not willing to buy cannabis in pharmacies, high disease burden that impede participation, fear of anonymity loss as cannabis is still prohibited in Switzerland, cannabis products do not meet participants’ needs or prices are too expensive compared to the illicit market, especially for heavy users. Moreover, the control group may have a high dropout rate, since this group may not be willing to wait 6 months until they get access. Secondly, the present study will not allow to draw any conclusions on the impact of participants’ cannabis use on others, e.g., family members, minors, or unrelated persons, nor on the illicit cannabis market due to the small sample size. Third, the majority of measurements will be self-reported that may be biased (recall bias, social desirability). However, pharmacies need to register all cannabis products they sell. Therefore, cannabis use is not only assessed as self-report, but also objectively assessed by pharmacists.

In conclusion, the present randomized controlled trial on regulated cannabis access for recreational use in Switzerland may contribute to identify a cannabis regulatory approach that balances between potential risks and benefits for all and may inform current and future public health efforts.

## Ethics statement

The study has been approved by the local ethics committee “Ethikkommission Nordwest- und Zentralschweiz” (Ethics Committee Northwest and Central Switzerland) as well as by the Swiss Federal Office of Public. The patients/participants will provide their written informed consent to participate in this study.

## Author contributions

MW is the principal investigator of the study, was responsible for the epigenetic analyses, and was involved in the eligibility screenings. LB-F is co-investigator and wrote the first draft of the manuscript. MW, LB-F, and RS were involved in the development and implementation of the study “Weed Care.” MW, RS, MM, and MV reviewed the manuscript. All authors contributed to the article and approved the submitted version.

## Funding

The study received funding by the canton Basel-Stadt. The funding had no role in study design and preparation of this manuscript, and will not have any role in data collection, analysis, and interpretation of the data.

## Conflict of interest

The authors declare that the research was conducted in the absence of any commercial or financial relationships that could be construed as a potential conflict of interest.

## Publisher’s note

All claims expressed in this article are solely those of the authors and do not necessarily represent those of their affiliated organizations, or those of the publisher, the editors and the reviewers. Any product that may be evaluated in this article, or claim that may be made by its manufacturer, is not guaranteed or endorsed by the publisher.

## Abbreviations

ASRS-V1.1: Adult ADHD Self-Report Scale
ASSIST: Alcohol, Smoking and Substance Involvement Screening Test
AUDIT-C: Alcohol Use Disorders Identification Test: CAT COPD: Assessment Test
CBD: Cannabidiol
CS-CCU: Comprehensive Short Questionnaire of Current Cannabis Use
CUDIT-R: Cannabis Use Disorders Identification Test-Revised
EKNZ: Ethikkommission Nordwest- und Zentralschweiz
EuroQol-5D: European Quality of Life
ERIraos: Early Recognition Inventory Checklist
GAD-7: Generalized Anxiety Disorder
GLTEQ: Godin Leisure Time Exercise Questionnaire
HPA axis: Hypothalamic–pituitary–adrenal axis
LRCU: Lower-Risk Cannabis Use
PANAS: Positive Affect and Negative Affect Scale
PHQ-9: Patient Health Questionnaire
PSQI: Pittsburgh Sleep Quality Index
REDCap: Research Electronic Data Capture
RCT: Randomized Controlled Trial
SAE: Serious Adverse Events
SCL-90: Somatization Symptom Checklist-90
S-CNU: Short Questionnaire of Current Nicotine Use
THC: Tetrahydrocannabinol
